# Radiographic imaging of the entheses of the equine thoracic foot

**DOI:** 10.1002/vetr.6024

**Published:** 2025-12-03

**Authors:** Donald Henre Honnas, Andrew R. Fiske‐Jackson, Caroline V. Fulkerson, D. Ray Wilhite

**Affiliations:** ^1^ Royal Veterinary College London UK; ^2^ Purdue University West Lafayette Indiana USA; ^3^ Texas Equine Hospital Bryan Texas USA; ^4^ Auburn University Auburn Alabama USA

**Keywords:** entheses, foot, horse, ligaments, radiographic anatomy, tendons

## Abstract

**Background:**

An injury of a tendon or ligament of the equine foot is frequently a cause of lameness. The optimal radiographic projections delineating the entheses of five commonly injured tendons and ligaments of the foot should be described.

**Methods:**

The entheses of the collateral ligaments of the distal interphalangeal joint, the collateral sesamoidean ligaments of the navicular bone, the deep digital flexor tendon, the common digital extensor tendon and the distal sesamoidean impar ligament were isolated from a thoracic foot of three cadavers. Three standard radiographic projections (60‐degree, dorsoproximal‒palmarodistal oblique [D60°PrPaDO] projection, lateromedial [LM] projection and dorsopalmar [DP] projection) were obtained of each foot, with the entheses of the selected tendons and ligaments identified with a marker.

**Results:**

The entheses of all structures analysed in this study were best identified on the D60°PrPaDO and LM projections. The insertion of the collateral ligaments of the distal interphalangeal joint was also easily identified on the DP projection.

**Limitations:**

A limitation was the inability to distinguish between lateral and medial sides of the feet.

**Conclusions:**

The radiographic images of the foot created in this study can serve as a guide for identifying enthesopathies of the ligaments and tendons of the equine foot.

## INTRODUCTION

Lameness of performance horses caused by pain in a foot is a common problem.^1^, ^2^, ^3^, ^4^, ^5^ After pain causing lameness has been isolated to a region of the limb by using diagnostic anaesthesia, radiography is usually the first diagnostic imaging modality used to identify a lesion causing pain in the region isolated.^4^, ^6^, ^7^ Ultrasonography is a useful diagnostic tool for examining soft‐tissue structures of the limbs, but transcuneal ultrasonographic examination provides adequate imaging of only the central structures of the distal aspect of the foot.^4^, ^8^, ^9^, ^10^ Structures medial, lateral and dorsal to the frog cannot be seen clearly ultrasonographically because sound waves cannot penetrate the hoof capsule.^4^, ^8^, ^9^, ^10^ More sophisticated imaging modalities, such as magnetic resonance imaging (MRI) and computed tomography (CT), provide images of bone and soft‐tissue structures superior to those provided by radiographs. Even though MRI and CT have become more accessible, these imaging modalities are still unavailable in many private practices, are usually far more expensive than conventional radiography, and often can be used only with the horse anaesthetised.^4^, ^5^, ^9^, ^11^, ^12^, ^13^, ^14^

**FIGURE 1:**
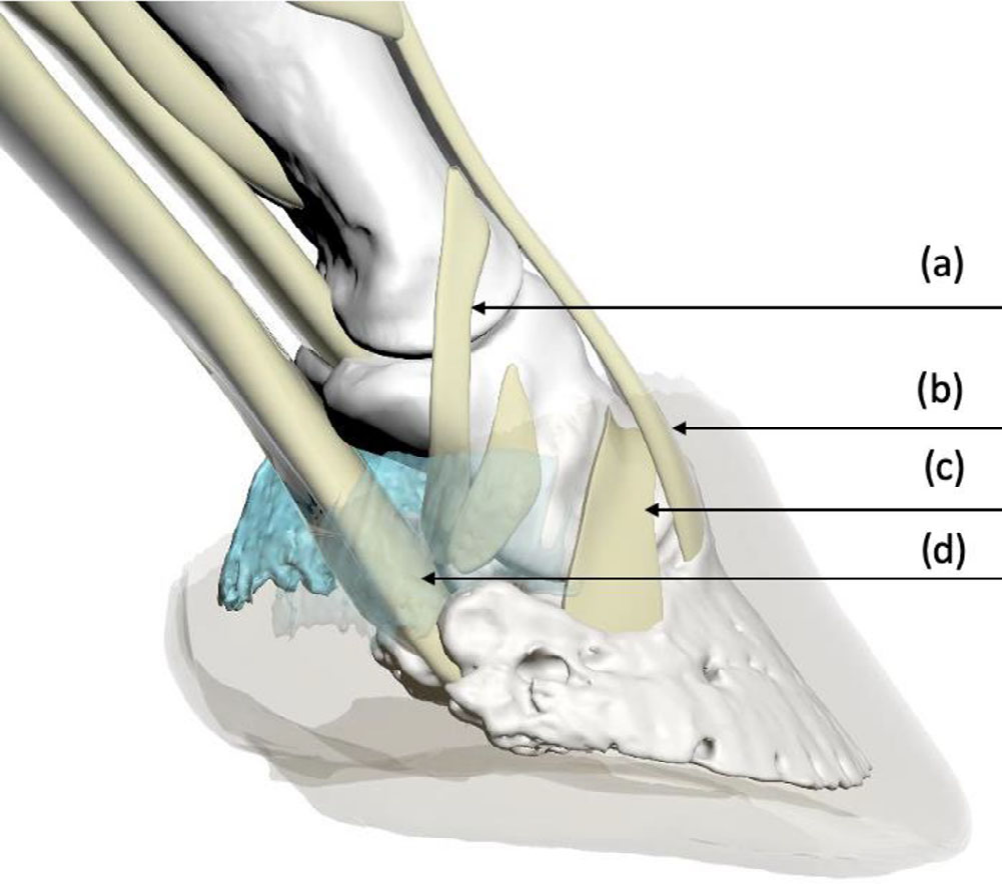
Three‐dimensional model illustrating the entheses of the collateral sesamoidean ligament (a); common digital extensor tendon (b); collateral ligament of the distal interphalangeal joint (c); and the deep digital flexor tendon (d). *Note*: Hoof capsule and collateral cartilage have been rendered partially transparent.

**FIGURE 2:**
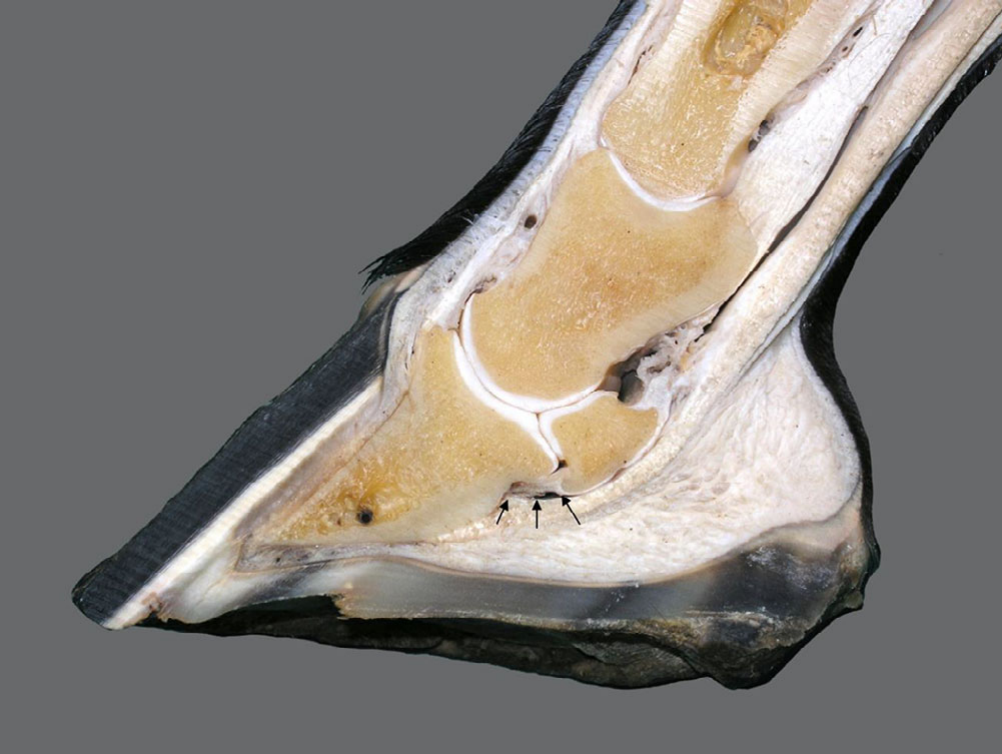
A transected foot depicting the distal sesamoidean impar ligament (arrows) and its entheses.

Lesions within the body of a tendon or ligament, except perhaps mineralisation, cannot be detected during radiographic examination of the equine foot, but when the origin or insertion of a tendon or ligament is injured, the injury may result in formation of new bone or demineralisation of bone at the site of injury that often can be detected radiographically.^4^, ^15^, ^16^, ^17^, ^18^ Knowing the radiographic location of the entheses of commonly injured ligaments and tendons of the foot (Figures 1 and 2) could allow clinicians to determine which ligament or tendon of the foot has been damaged when mineralisation, sclerosis, lysis or new bone formation is observed during evaluation of standard radiographs of the foot.^4^, ^18^

An objective of this study was to evaluate three standard radiographic projections of the equine foot, with a marker placed on the insertion of two tendons commonly injured within the foot and on the origin and insertion of three commonly injured ligaments of the foot, to determine which of the three standard projections of the foot best delineated the site of each enthesis or pairs of entheses. Another objective was to identify and describe radiographic landmarks associated with each enthesis, using the images that best delineated the site of each enthesis or pairs of entheses.^4^

## MATERIALS AND METHODS

**FIGURE 3:**
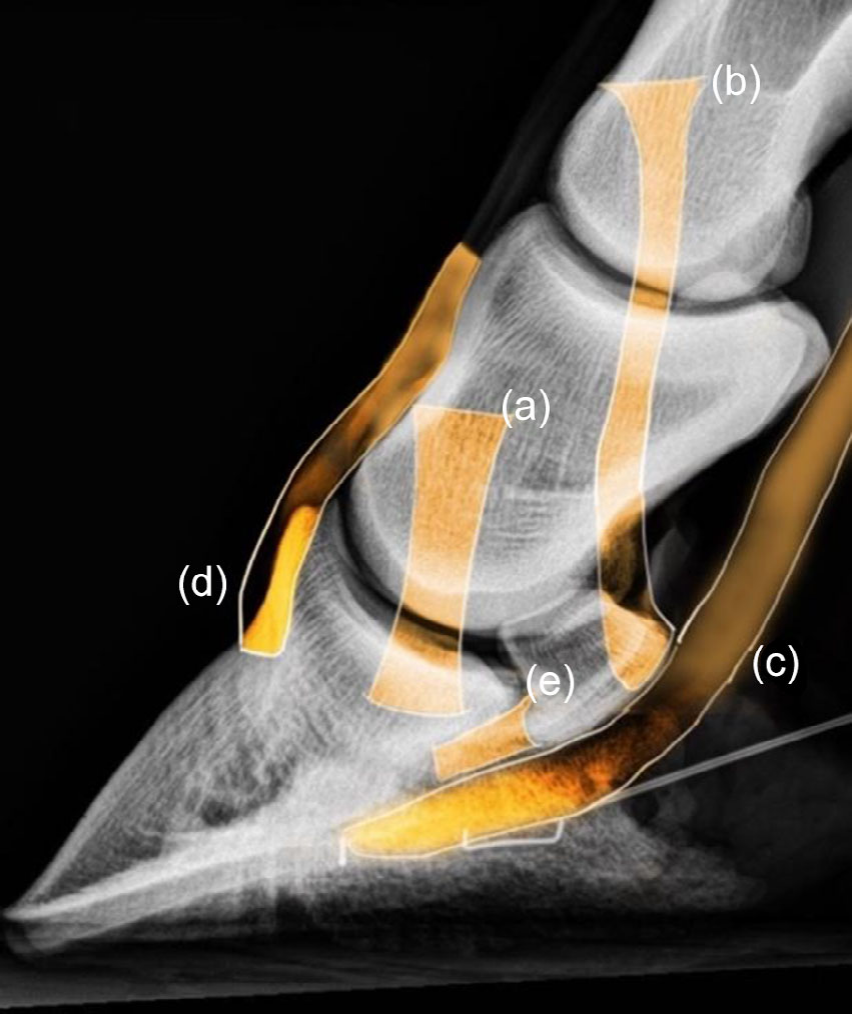
Radiographic guide (lateromedial projection) illustrating the entheses of the collateral ligaments of the distal interphalangeal joint (a); the collateral sesamoidean ligament (b); the deep digital flexor tendon (c); common digital extensor tendon (d); and the distal sesamoidean impar ligament (e).

Three thoracic digits harvested from three horses, euthanased for reasons unrelated to lameness, were dissected to expose the 12 entheses of each foot examined in this study. After removing the sole and dorsal aspect of the hoof with hoof nippers, the medial and lateral aspects of the hoof were separated from the foot. The entheses of the ligaments and tendons of interest were then exposed by dissection. The following entheses were exposed (Figure 3): the entheses of the origin and insertion of the medial and lateral collateral ligaments (CLs) of the distal interphalangeal (DIP) joint (four entheses); the entheses at the origin and insertion of the medial and lateral collateral sesamoidean ligaments (CSLs) of the navicular bone (four entheses); the enthesis of the insertion of the deep digital flexor tendon (DDFT; one enthesis); the enthesis of the insertion of common digital extensor tendon (CDET; one enthesis); and the entheses of the origin and insertion of the distal sesamoidean impar ligament (DSIL; two entheses).

A pair of entheses of the foot was defined to include the medial and lateral origins or the medial and lateral insertions of a paired ligament, so that each of the paired ligaments of the foot had two pairs of entheses; for example, the enthesis of the origin of the medial CL of the DIP joint and that of the lateral CL of the DIP joint were considered to be a pair of entheses. Both pairs of enthesis of a paired ligament of each foot were marked with a surgical staple or a 23‐gauge needle, so that four entheses (two origins and two insertions) were tagged with a marker.

The following three standard radiographic projections were obtained with a portable X‐ray unit (Machine Model: ULTRA 9020BT, Ecoray Co., Ltd., 3F Urbanlight B/D) for each enthesis or pairs of entheses, of each tendon or ligament of interest, by using a kVp of 60 and an mAs of 0.12: the 60‐degree, dorsoproximal‒palmarodistal oblique (D60°PrPaDO) projection; the lateromedial (LM) projection; and the dorsopalmar (DP) projection. Each projection was examined to determine which of the three standard projections best delineated the site of each enthesis or pairs of entheses. For example, to determine which projection best imaged the entheses of the origin and insertion of the paired CLs of the DIP joint, the origin and insertion of each CL were marked, and DP projection and D60°PrPaDO projections were obtained. For all LM projections, the markers at the origin and insertion of one of the paired CLs of the DIP joint and the CSLs of the navicular bone were removed to reduce superimposition of the markers.

For enthesis at the origin and the enthesis at the insertion of the DSIL, the enthesis at the insertion of the CDET, and the enthesis at the insertion of the DDFT, the DP projection, and a D60°PrPaDO projection were obtained with a marker on both sides of the structure, but for the LM projection the marker was removed from one side of the structure to avoid superimposition of markers. For example, to determine which projection best imaged the enthesis at the insertion of the unpaired DDFT, the medial and lateral aspects of the DDFT were each marked, and DP and D60°PrPaDO projections were obtained. To obtain the LM projection, however, a marker was removed from one side of the DDFT at the tendon's insertion to avoid superimposition of markers.

The entheses of each of the five anatomical structures (CDET, DDFT, CSL, DSL and CLs of the DIP joint) were marked separately, as described above, and radiographed using three projections for each anatomical structure, yielding 15 radiographic images per foot. Therefore, 45 images obtained from three feet were examined to determine which of three standard images of the foot best showed each enthesis or group of entheses.

A board‐certified veterinary surgeon and a board‐certified veterinary radiologist determined independently which image(s) best delineated each enthesis or group of entheses. Using the selected radiographs, the landmarks that could be used to locate each enthesis radiographically were described.

## RESULTS

**FIGURE 4:**
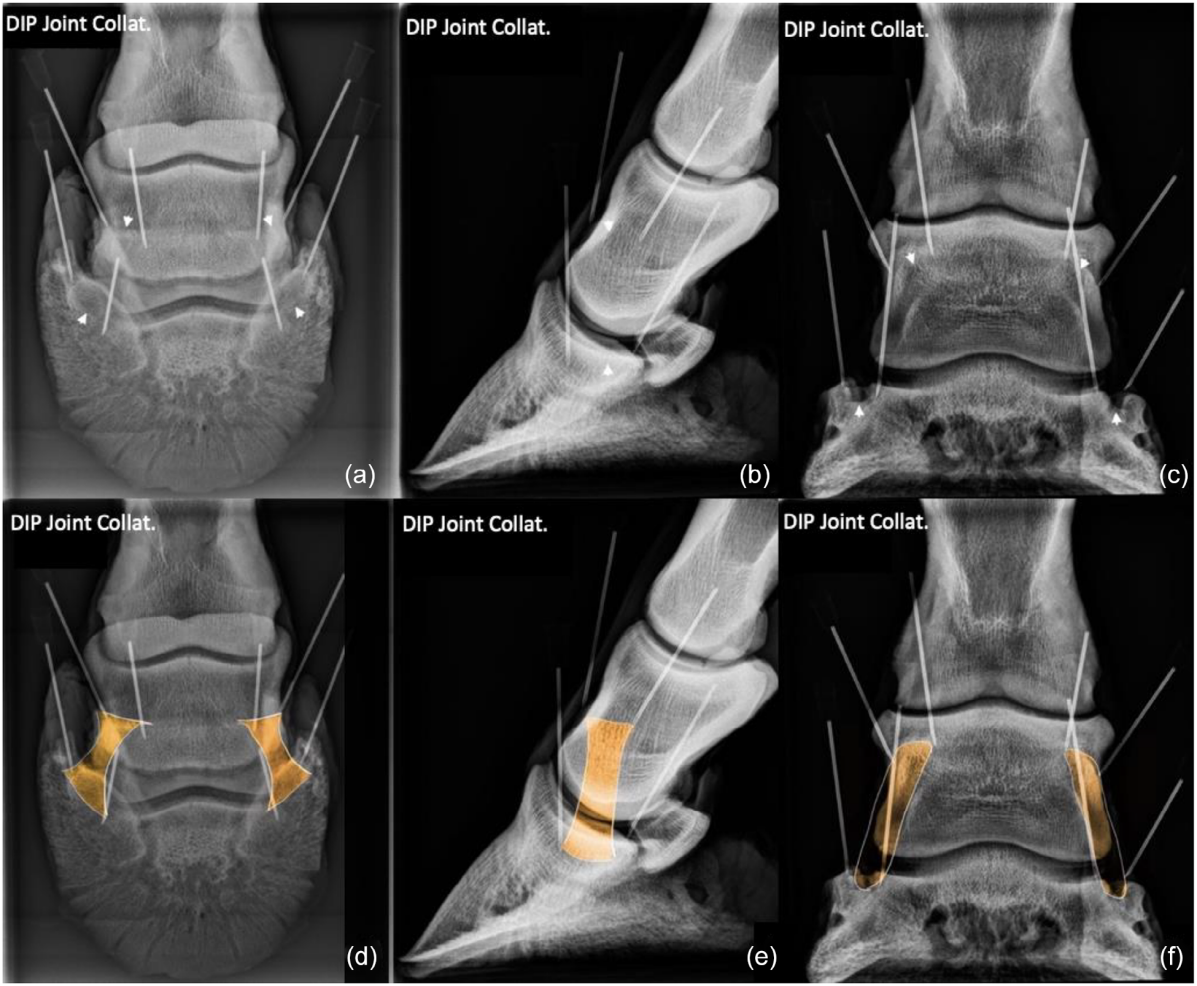
Collateral ligaments (CLs) of the distal interphalangeal (DIP) joint: CLs of the DIP joint originate at a depression, one on each side of the dorsodistal aspect of the middle phalanx (proximal arrows). The ligament crosses a depression in the distal aspect of the middle phalanx and attaches at a depression on the proximal aspect of the distal phalanx (distal arrows). The origin of both the lateral and medial ligaments are best viewed in the 60‐degree, dorsoproximal‒palmarodistal oblique (D60°PrPaDO) (a and d) and lateromedial (b and e) projections. The insertions are best viewed in the D60°PrPaDO (a and d) and dorsopalmar (c and f) projections. The origins and insertions of the lateral and medial ligaments are superimposed in the lateromedial projection.

**FIGURE 5:**
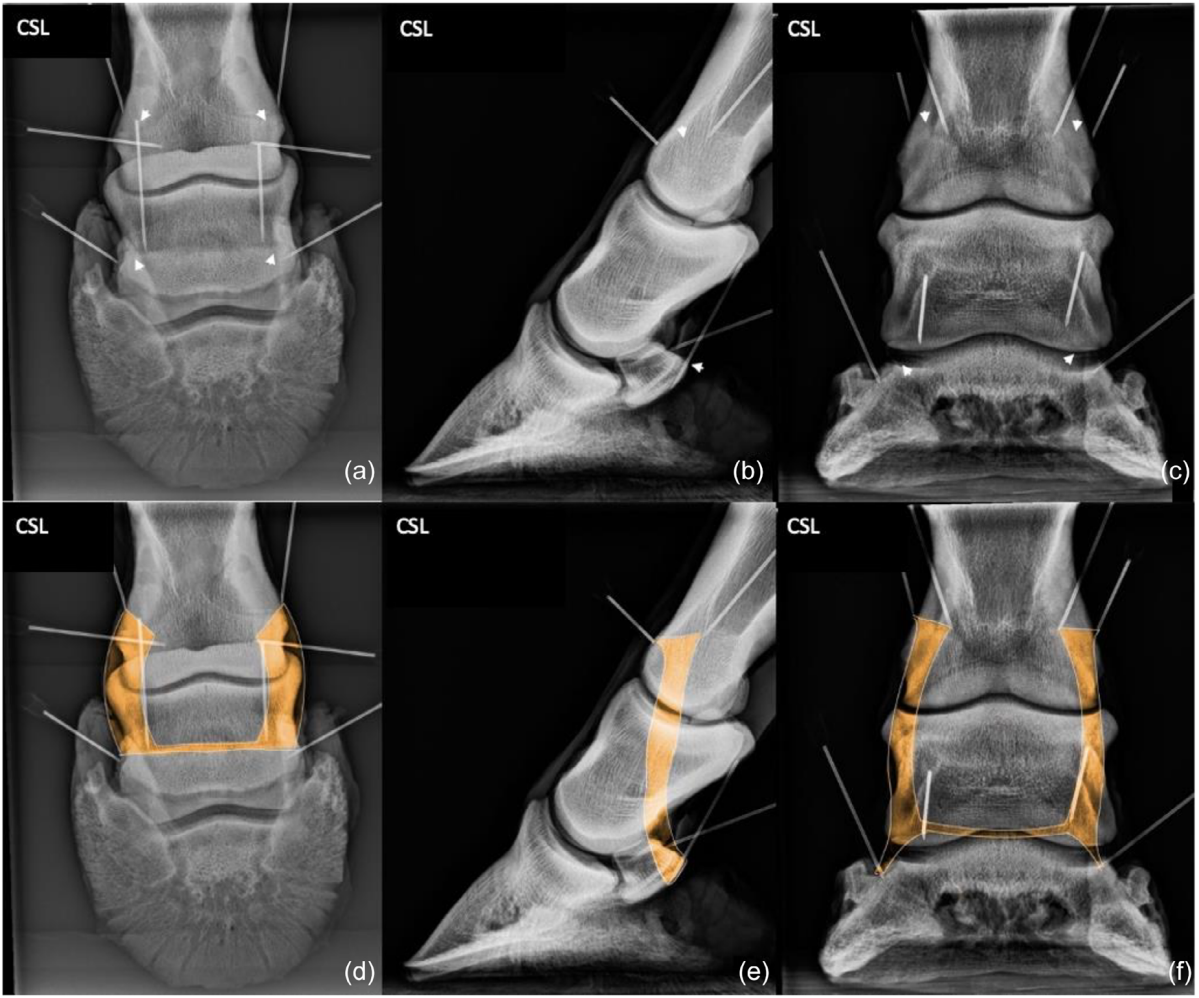
Collateral sesamoidean ligament (CSL): medial and lateral CSLs of the navicular bone attach to the portion of the ligamentous complex that originates from a depression on each side of the distal aspect of the proximal phalanx (proximal arrows). The CSLs extend distopalmarly and insert on the extremities (wings) and proximal border of the navicular bone (distal arrows) to suspend the navicular bone. The entheses at the origin and insertion of the paired ligament are identified by small areas of increased radiodensity best viewed on the 60‐degree, dorsoproximal‒palmarodistal oblique (a and d) and lateromedial (b and e) projections.

**FIGURE 6:**
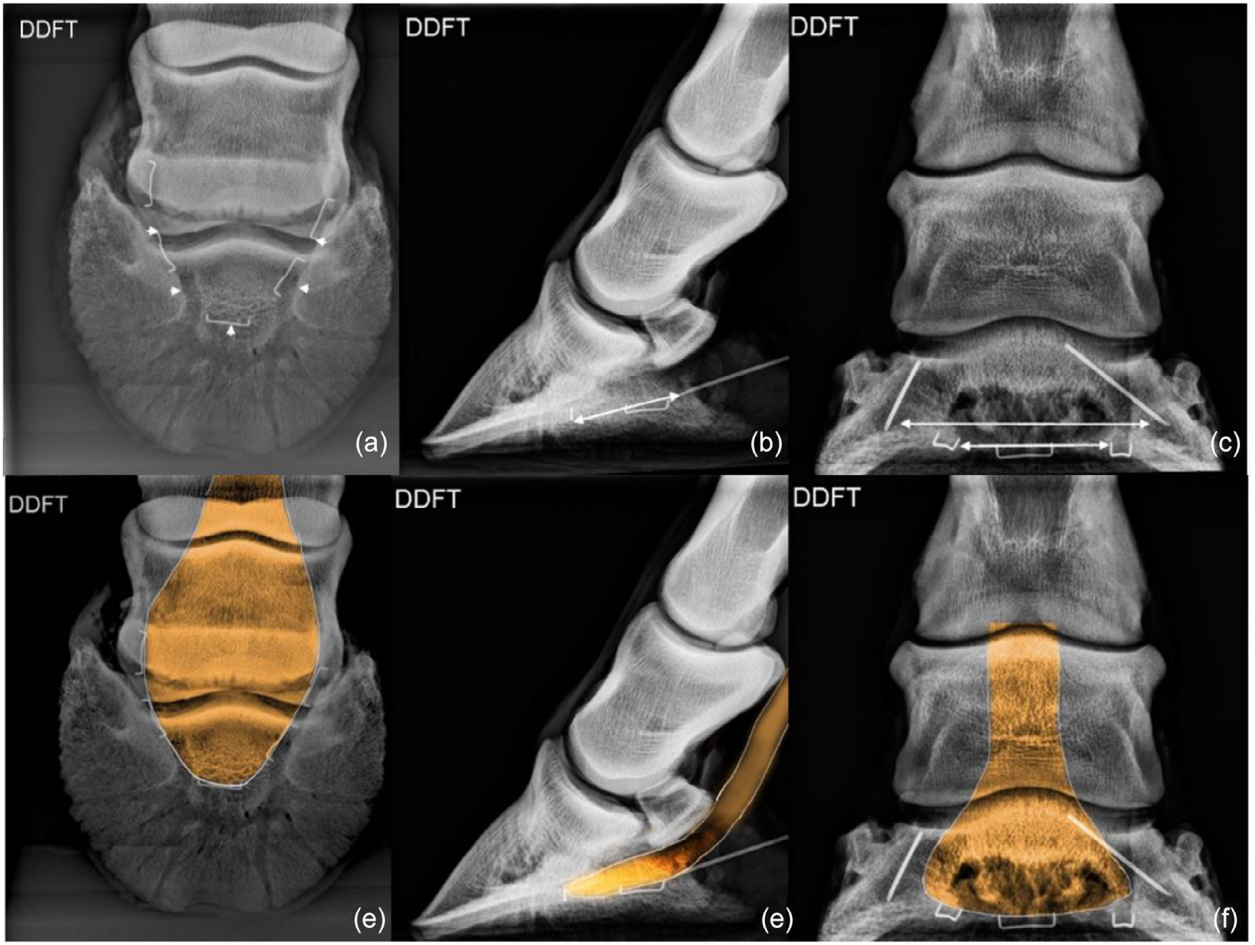
Deep digital flexor tendon (DDFT): DDFT inserts on the facies flexoria of the distal phalanx (arrows). The insertion is best viewed on the 60‐degree, dorsoproximal‒palmarodistal oblique (D60°PrPaDO) (a and d) and lateromedial (b and e) projections. The dorsopalmar projection provides a poor image of the insertion, and superimposition of the site of insertion with the distal phalanx and distal aspect of the navicular bone was greater than that seen in the D60°PrPaDO projection.

**FIGURE 7:**
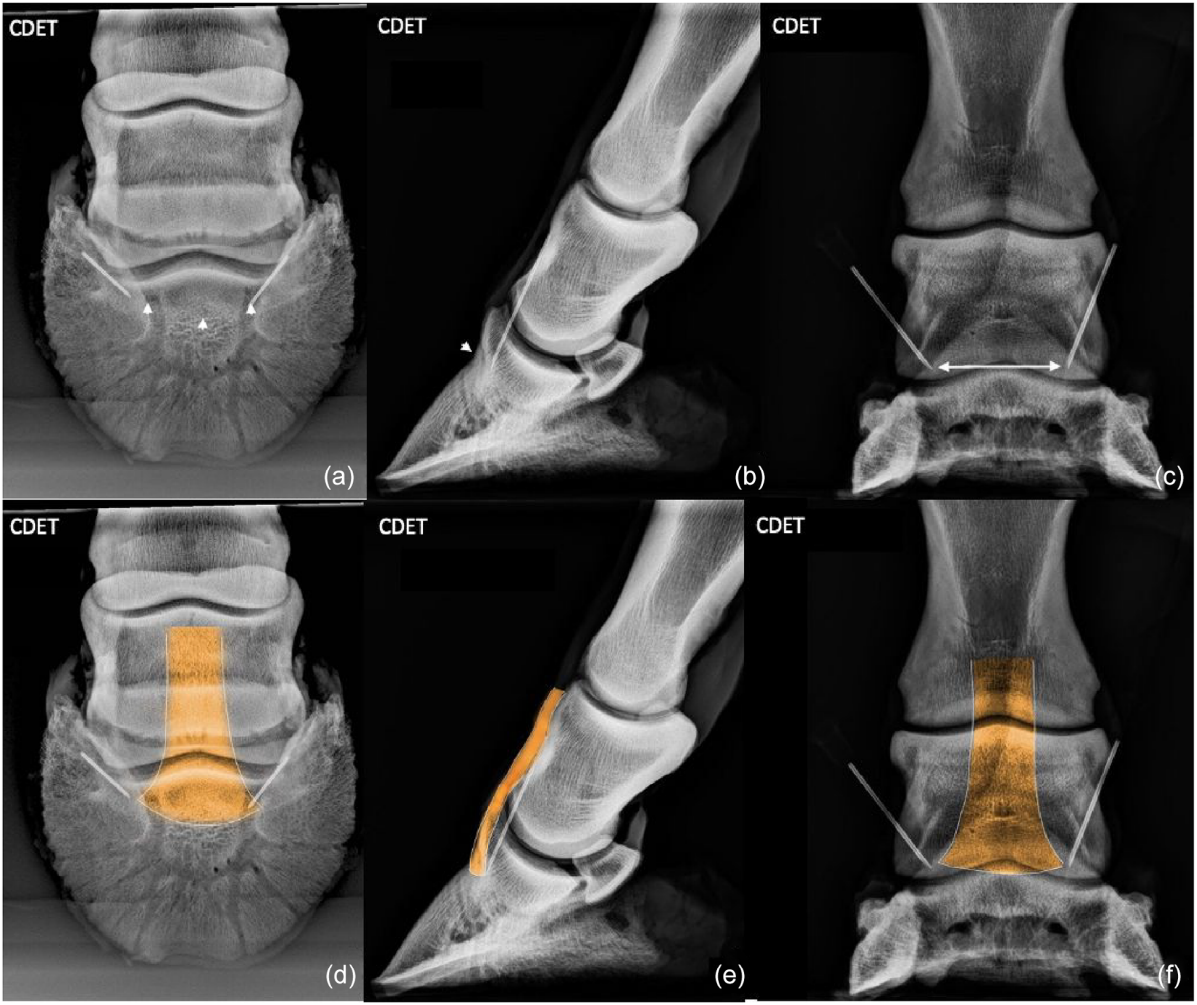
Common digital extensor tendon (CDET): CDET inserts on the extensor process of the distal phalanx (arrows) and was best viewed on the 60‐degree, dorsoproximal‒palmarodistal oblique (a and d) and lateromedial (b and e) projections. The site of insertion is obscured on the dorsopalmar projection (c and f) by the superimposed distal aspect of the middle phalanx.

**FIGURE 8:**
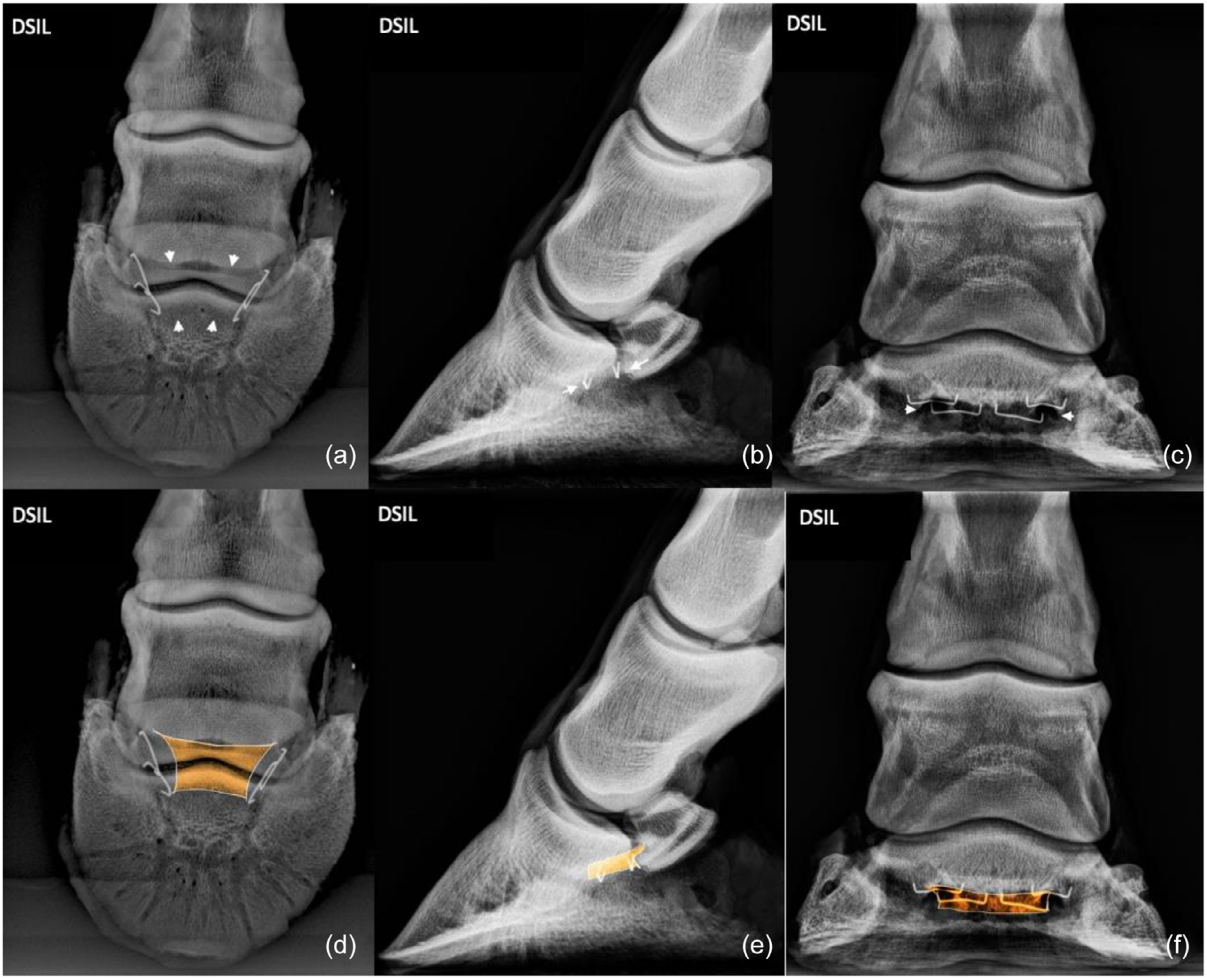
Distal sesamoidean impar ligament (DSIL): DSIL originates on the distopalmar aspect of the navicular bone (proximal arrows) and inserts distally at the palmar margin of the solar surface of the distal phalanx (distal arrows). The origin and insertion of the ligament are best viewed on the 60‐degree, dorsoproximal‒palmarodistal oblique (a and d) and lateromedial (b and e) projections. The dorsopalmar projection (c and f) provides a poor image of the insertion, and the site of insertion is superimposed by the distal phalanx and distal aspect of the navicular bone.

The results of our dissection demonstrated the extensive interconnectivity of the ligaments of the equine foot. The three standard radiographic projections for the 12 entheses are shown in Figures 4, 5, 6, 7, 8. No difference was found in outcome between the surgeon and radiologist in selection of images that best delineated the individual or paired entheses examined in this study, and no difference was found between images selected for each of the three feet used in this study. The radiographic projections that show changes indicative of an enthesopathy are available as Supporting Information S1‒S5. The radiographic landmarks for identifying each enthesis are provided in Table 1.

**TABLE 1:** Summary of the radiographic anatomy of the entheses of the foot.

Structure	Origin	Insertion	Optimal radiographic projections	Radiographic landmarks
Collateral ligaments of the distal interphalangeal joint	Fossa on the dorsodistal aspect of middle phalanx	Collateral fossa of the distal phalanx	D60°PrPaDO LM	Origin: immediately proximal to the proximal border of the navicular bone. Insertion: in the collateral fossa located on the dorsomedial and dorsolateral aspects at the proximal end of the distal phalanx. Origin: fossa on dorsodistal aspect of the middle phalanx. Insertion: radiolucent depression on the proximal aspect of the distal phalanx.
Collateral sesamoidean ligaments of the navicular bone	Distolateral/distomedial aspects of the proximal phalanx	Proximal border of the navicular bone	D60°PrPaDO LM	Origin: radiolucent depression on the proximo‐lateral, and proximo‐medial aspects of the distal condyles of the proximal phalanx. Insertion: on the extremities/wings and proximal border of the navicular bone. Same as for the D60°PrPaDO. This projection shows the dorsally located radiolucent depressions at the origin of each collateral sesamoidean ligament on the proximal phalanx and the distopalmar orientation towards the proximal border of the palmar navicular compact bone.
Deep digital flexor tendon	Deep digital flexor muscle on the medial humeral epicondyle radius and ulna	Facies flexoria of the distal phalanx	D60°PrPaDO LM	Insertion: approximately in the centre of the facies flexoria of the distal phalanx. Insertion: palmar aspect of solar surface of distal phalanx. Near centre of the facies flexoria.
Common digital extensor tendon	Common digital extensor muscle on the lateral humeral epicondyle	Extensor process of the distal phalanx	D60°PrPaDO LM	Insertion: at the base of the extensor process. An increase in osseous radiodensity reveals the site of insertion. Insertion: dorsal aspect of the base of the extensor process, rather than the apex.
Distal sesamoidean impar ligament	Distal border of the navicular bone	Adjacent to the facies flexoria on the palmar aspect of the solar surface	D60°PrPaDO LM	Origin: distal border of navicular bone. Insertion: adjacent to facies flexoria of the distal phalanx. Origin: distopalmar aspect of the navicular bone. Insertion: palmar aspect of solar surface of distal phalanx.

Abbreviations: DP, dorsopalmar; D60°PrPaDO, dorso60°proximal‒palmarodistal oblique; LM, lateromedial.

### Collateral ligaments of the DIP joint

The origins and insertions of CLs of the DIP joint were best viewed on the D60°PrPaDO projection. The origin and insertion of each CL were superimposed with its counterpart on the LM projection. The insertion was easily identified on the DP projection.

### Collateral sesamoidean ligaments

The origin and insertion of the paired CSL were best viewed on the D60°PrPaDO and LM projections. The D60°PrPaDO and LM projections provided good images of the origin and insertion of each ligament, but the origin and insertion of each CSL were superimposed with their counterparts on the LM projection. Superimposition of the middle and distal phalanx on the insertion of the CSLs on the navicular bone was more pronounced on the DP projection than on the other projections.

### Deep digital flexor tendon

The insertion of the DDFT was best viewed in the D60°PrPaDO projection and LM projections. The DP projection provided a poor image of the insertion, and superimposition of the site of insertion with the center of the distal phalanx and distal aspect of the navicular bone was greater than that seen in the D60°PrPaDO projection.

### Common digital extensor tendon

The enthesis at the insertion of the CDET was best viewed on the D60°PrPaDO and LM projections. The insertion was readily visible on the D60°PrPaDO projection because the angle of the projection allowed the broad‐based site of insertion to be imaged on the extensor process of the distal phalanx without superimposition of the middle phalanx. The site of insertion was obscured on the DP projection by the superimposed distal aspect of the middle phalanx.

### Distal sesamoidean impar ligament

The origin and insertion of the DSIL were slightly more lucent than the surrounding bone and were best viewed on the D60°PrPaDO and LM projections. The DP projection provided a poor image of the site of insertion because the site of insertion was superimposed by the distal aspect of the navicular bone.

## DISCUSSION

An enthesopathy appears radiographically as an enthesophyte, erosion, mineralisation or hyperostosis where a tendon or ligament originates or inserts at a bone.^19^ The origin of a ligament is the end that attaches to a structure that is more stable than the structure to which the ligament inserts. The origin of a ligament is usually located proximal to the insertion of the ligament.^20^

Radiographic identification of the origin and insertion of the tendons and ligaments of the equine stifle was described by Aldrich et al.,^4^ and Casillas et al.^21^ radiographically identified the origins and insertions of the tendons and ligaments of the equine tarsus, but we can find no published radiographic descriptions of the origins and insertions of the tendons and ligaments of the equine foot.

The standard radiographic projections of foot that best showed the entheses of five commonly injured tendons and ligaments of the equine digit were identified to serve as a guide for identifying enthesopathies of the ligaments and tendons of the equine foot.

The D60°PrPaDO projection of the distal phalanx best showed the radiolucent areas, medially and laterally, that are the sites of insertion of the CLs of the DIP joint.^22^ Radiographic osseous changes of the foot, such as sclerosis, lucency or new bone forming an enthesophyte, seen at the site of origin or insertion of a ligament or tendon should prompt further evaluation in other projections (such as the dorsal 60° lateral‒palmaromedial oblique and dorsal 60° medial‒palmarolateral oblique projections) because an enthesopathy may become more apparent in these projections. Oblique views were not included in this study because when a horse is examined radiographically to determine the cause of lameness that has been localised to the foot, the LM, DP and D60°PrPaDO are usually the first radiographic projections obtained; other projections selected are based on abnormalities noted during examination of those projections. When a lesion or suspected lesion is observed on one of these three projections, other radiographic projections should be obtained to allow a geometrical, tridimensional reconstruction of the lesion. Many soft‐tissue lesions of the equine foot do not involve entheses; however, in the absence of radiographic evidence of ligamentous or tendinous damage, cross‐sectional imaging (MRI or CT) may be indicated to determine what structure has been damaged.

The CLs of the DIP joint originate at a depression on the middle phalanx, one on each side of the dorsodistal aspect of the phalanx.^22^, ^23^ The ligament crosses a depression in the distolateral aspect of the middle phalanx and inserts in a radiolucent depression on the proximolateral aspect of the distal phalanx.^8^ Injury to a CL of the DIP joint may result in formation of a radiographically evident enthesophyte at the site of the origin of the CL on the dorsodistal aspect of the middle phalanx.^22^ Enthesopathy may also appear at the insertion of the CL as a focal lucency or as an enlargement of the fossa of the distal phalanx.

An enthesopathy at the origin or insertion of the CL of the DIP joint is not always clinically significant, but it may indicate that the ligament has suffered acute or chronic trauma.^22^ Dyson and Murray suggested that many fractures of the palmar process of the distal phalanx occur immediately palmar to the insertion of the CL of the DIP joint and that injury to the ipsilateral CL of the DIP joint may create instability of the DIP joint, which could contribute to delayed healing.^24^ They reported that some horses acutely lame because of fracture of a palmar process have radiographic changes in the trabecular pattern in the bone bordering the fracture, indicating that the fracture may be the manifestation of repetitive ligamentous strain propagated through the insertion of the ipsilateral CL of the DIP joint.^24^

The origin and insertion of the CSLs of the navicular bone are complex; therefore, the attachments focused on in this study were the suspensory portions of the ligamentous complex. The CSL originates at a radiolucent depression on the distomedial/distolateral aspect of the proximal phalanx.^25^, ^26^ The medial and lateral CSLs extend palmarodistally to insert on the extremities (wings) and proximal border of the navicular bone.^25^, ^26^ Each CSL originates dorsal to the CL of the proximal interphalangeal joint and extends distopalmarly along the lateral border of the proximal and middle phalanges to its extensive insertion on the proximal border of the navicular bone. Lesions of the CSL can sometimes be seen, by using MRI, in association with abnormal mineralisation extending from the cortex of the proximal border of the navicular bone, at the bone's extremities, into the spongiosa.^3^

The DSIL originates from Sharpey's fibres on the distopalmar aspect of the navicular bone and inserts distally at the palmar margin of the solar surface of the distal phalanx.^27^, ^28^ Lesions of the DSIL can sometimes be seen, by using MRI along the entire distal border of the navicular bone in association with fragments and with abnormal mineralisation extending from the cortex into the spongiosa. These abnormalities may be a chronic response to pathological stress of the podotrochlear apparatus.^3^ Small radiopacities (enthesopathies) can often be seen in the DSIL distal to the navicular bone of some horses. These radiopacities may be avulsion fractures of the navicular bone or dystrophic mineralisation of the DSIL.^15^, ^27^, ^29^, ^30^ The D60°PrPaDO and LM projections were the most informative for assessing the entheses of the DSIL and, therefore, are the best projections for observing enthesopathies associated with the ligament.

The DDFT inserts in a smooth concavity on the distal phalanx termed the facies flexoria.^31^, ^32^ The cortex of the distal phalanx at this location should appear smooth and regular radiographically where it meets the more proximal site of the insertion of the DSIL.^22^ Tearing of the DDFT at its insertion at the facies flexoria has been reported to result in production of irregular new bone, which may be visible radiographically,^22^ but the experience of the authors and that of others (correspondence with Michael Schramme) suggests that radiographic evidence of damage to the DDFT at its insertion is rare, if ever observed. Resorption of the flexor surface of the distal phalanx, however, is not uncommon and can be identified by MRI and, in many cases, also by radiographic examination.^33^ This resorption is often associated with injury to the DDFT and/or navicular bone. Transcuneal, ultrasonographic examination may provide additional information about the severity of damage to the DDFT, but transcuneal ultrasonography requires intensive preparation of the frog, and the quality of images is poor. The D60°PrPaDO and LM projections were found to be the most useful for locating the enthesis at the insertion of the DDFT and assessing for the presence of enthesopathies at this site.

The CDET inserts on the extensor process of the distal phalanx.^31^ Tearing of the insertion of the CDET on the extensor process of the distal phalanx can occasionally result in lameness and formation of new bone on the dorsodistal aspect of the extensor process, rather than at the apex of the process.^22^ Rarely, an avulsion fracture can occur where the CDET inserts on the extensor process, resulting in a bone fragment displaced proximally by traction of the tendon.^18^ The D60°PrPaDO and LM projections were the most useful for imaging the enthesis at the insertion of the CDET.

We believe the projections displayed in this manuscript can be a useful reference for clinicians evaluating radiographs of the equine foot to identify an enthesopathy or an avulsion fracture at an enthesis.^4^ Although diagnosing an avulsion fracture or an enthesopathy radiographically is often possible, there are limiting factors to this imaging modality. Like formation of an osteophyte, formation of an enthesophyte can often take weeks before it can be identified radiographically,^18^ and an enthesophyte is not always associated with lameness.^18^ Acute damage to the origin or insertion of a ligament or the insertion of a tendon of the foot, therefore, might be better identified by using cross‐sectional imaging or nuclear scintigraphy, rather than radiography.^4^ The mineral density of a bone must change approximately 50% before a change in density can be identified radiographically.^4^, ^34^ As the ligamentous or tendinous injury becomes chronic, with repeated straining/spraining, mineralisation or radiolucency forms, creating an irregular margin at the enthesis.^18^ The density of the hoof wall, and the low maximum kVp of the conventional portable radiographic equipment present a substantial challenge for producing high‐definition radiographs suitable for identifying early, subtle changes in bone density at an enthesis. By using modern radiographic technology, however, high‐quality images can be produced that allow detection of subtle osseous changes; therefore, a detailed knowledge of the locations of the entheses of the foot is necessary to effectively evaluate the foot radiographically.^4^ This research should assist clinicians in diagnosing an injury to the origin or insertion of a ligament or insertion of a tendon of the foot.

### Limitations

Limitations of the study include unknown lameness status of the horses used in this study, and no knowledge of lateral versus medial side of the limb. Although the sample size was small, the sites of entheses are uniform among horses^31^, ^32^ and no radiographic or gross evidence of disease of the digits was identified. The lameness status was unknown, but the horses were euthanased for reasons unrelated to lameness. Because the right and left halves of the feet are mirror images of each other,^31^ we believe that knowledge of lateral versus medial was not necessary.

## AUTHOR CONTRIBUTIONS

Donald Henre Honnas and Andrew R. Fiske‐Jackson were responsible for overall study design. Manuscript was prepared by Donald Henre Honnas and revised by Andrew R. Fiske‐Jackson. Analysis of the radiographs and manuscript revision was performed by Caroline V. Fulkerson. Revision of manuscript and creation of the 3D models of the tendons and ligaments were performed by D. Ray Wilhite. Special thanks to Jim Schumacher for assisting with manuscript revision. All authors approved the final draft of the manuscript prior to submission.

## CONFLICT OF INTEREST STATEMENT

The authors declare they have no conflicts of interest.

## FUNDING INFORMATION

The authors received no specific funding for this work.

## ETHICS STATEMENT

The Clinical Research Ethical Review Board of the Royal Veterinary College in London granted ethical approval for this project (reference no. CR2019‐011‐2). The study was performed on material collected during postmortem examination. Explicit owner informed consent for participation in this study was not stated but general permission for postmortem examination and use of tissue in research was given.

## Supporting information



Supporting Information

Supporting Information

Supporting Information

Supporting Information

Supporting Information

## Data Availability

The data (radiographs) generated and analysed in this study that best delineated the entheses are provided in the main document, and clinical examples of enthesiopathy are provided in the Supporting Information. The remainder of the radiographs are available upon request from the authors.
