# Supplementary material for: Radiographic imaging of the entheses of the equine thoracic foot
Source: Vet Rec. 2025 Dec 3;198(4):e166–75. doi: 10.1002/vetr.6024 (PMC12904081; doi:10.1002/vetr.6024)
Supplement: Supplementary file 1 — Supporting Information [file VETR-198--s004.docx]

|  |
| --- |
| 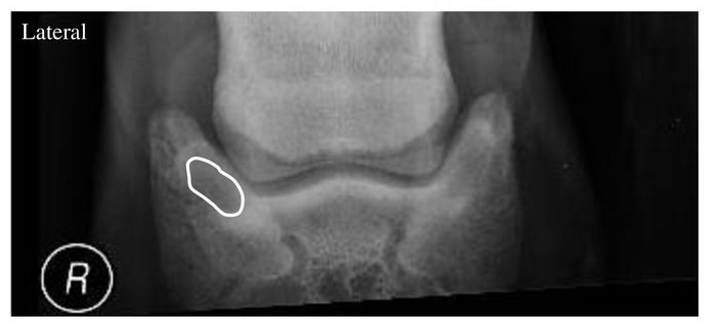 |
| Supplementary item 1: Example of enthesopathy at the insertion of the CL. The white outline denotes mild enlargement of the lateral collateral fossa of the distal phalanx with focal lucency at the insertion of the lateral CL of the DIP joint. Image reproduced from Axiak and Werpy [36], with permission. |
