# Supplementary material for: Radiographic imaging of the entheses of the equine thoracic foot
Source: Vet Rec. 2025 Dec 3;198(4):e166–75. doi: 10.1002/vetr.6024 (PMC12904081; doi:10.1002/vetr.6024)
Supplement: Supplementary file 2 — Supporting Information [file VETR-198--s001.docx]

| Radiographic projections showing the entheses of the equine foot |
| --- |
| 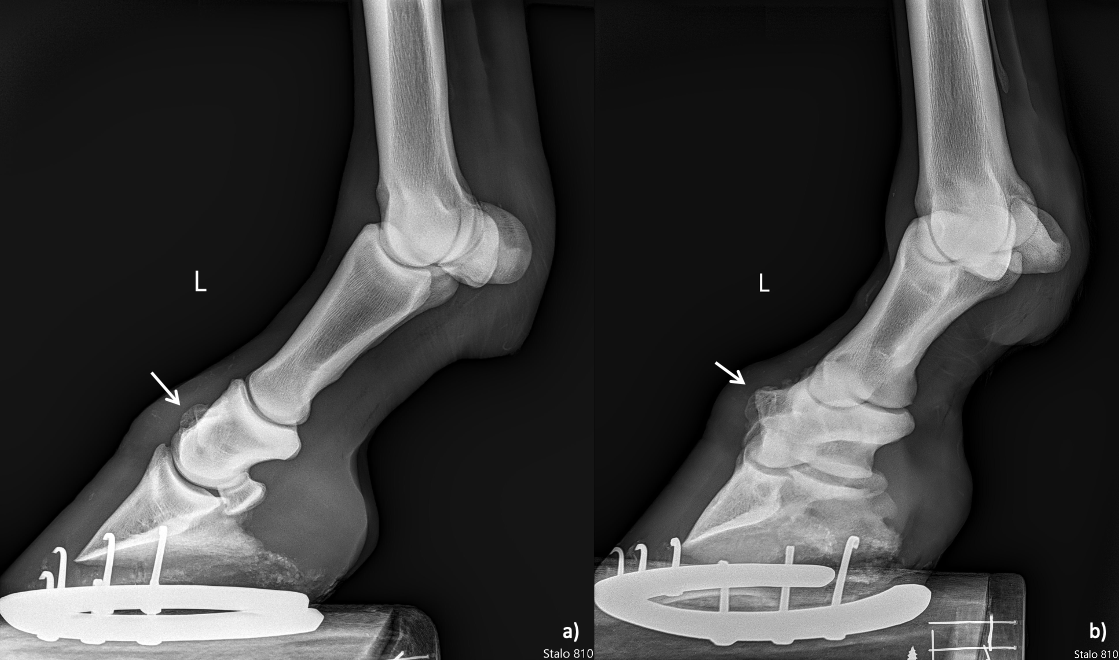 |
| Supplementary item 2: Example of enthesopathy of the origin of the collateral ligament of the distal interphalangeal joint - Courtesy of Texas Equine Hospital, with permission. |
