# Supplementary material for: Radiographic imaging of the entheses of the equine thoracic foot
Source: Vet Rec. 2025 Dec 3;198(4):e166–75. doi: 10.1002/vetr.6024 (PMC12904081; doi:10.1002/vetr.6024)
Supplement: Supplementary file 3 — Supporting Information [file VETR-198--s005.docx]

|  |
| --- |
| 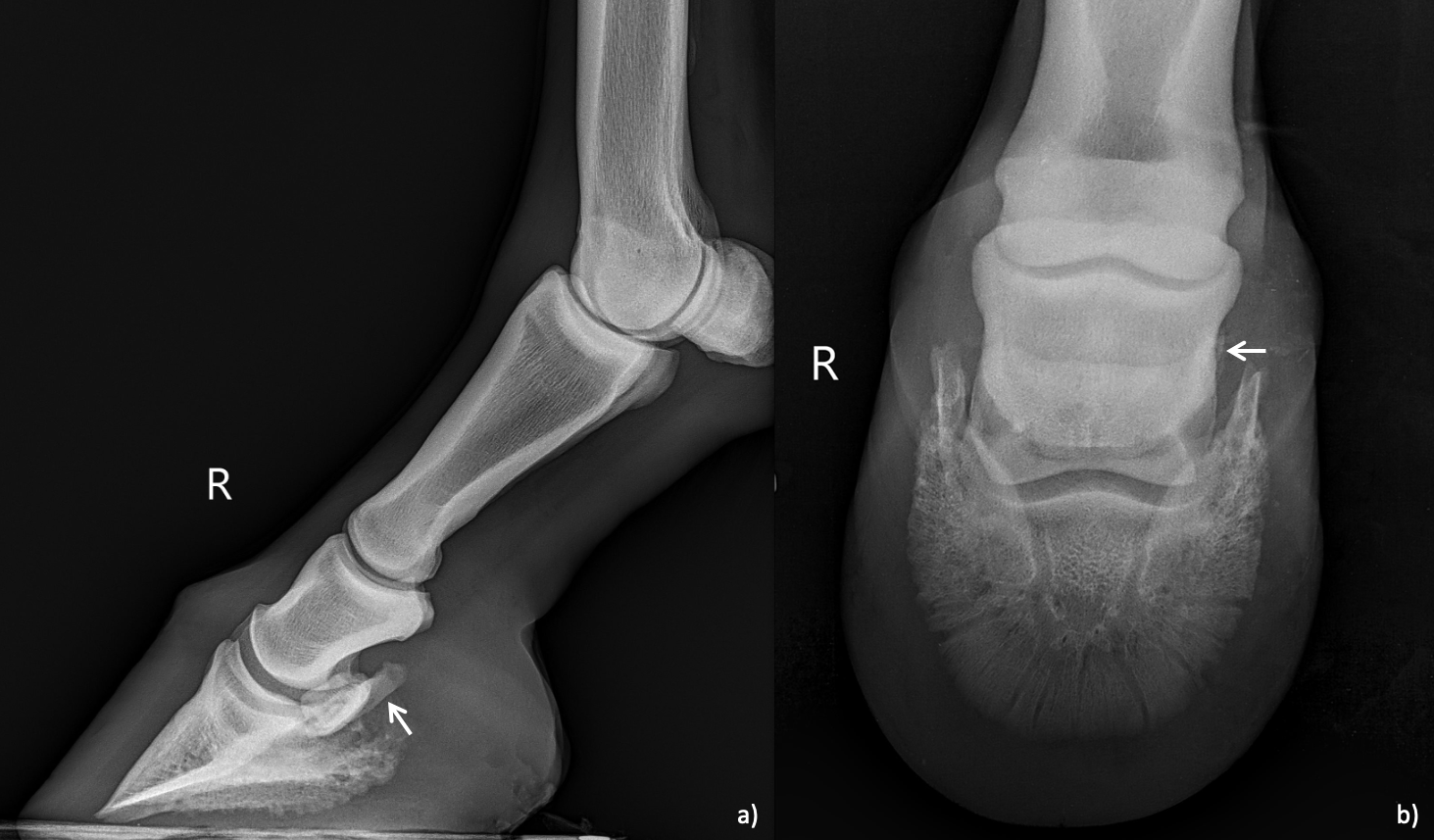 |
| Supplementary item 3: Example of enthesopathy of the collateral sesamoidean ligament insertion - Courtesy of Texas Equine Hospital, with permission. |
